# A peripheral proteomic signature of Alzheimer’s disease is identified in the plasma extracellular vesicles of mild cognitive impairment patients from a memory clinic: the BIOPEXAL study

**DOI:** 10.21203/rs.3.rs-7847549/v1

**Published:** 2025-11-01

**Authors:** Maria Capdevila-Bayo, Rosanna Rossi, Itziar de Rojas, Raquel Puerta, Laura Guzmán, Marina Carrasco, Álvaro Muñoz-Morales, Claudia Olivé, Laura Montrreal, Pablo García-González, Paula Bayón-Buján, Andrea Miguel-Romero, Berta Calm, Oscar Sotolongo-Grau, Adelina Orellana, Natalia Tatinya, Marta Martínez, Montserrat Alegret, Pilar Sanz-Cartagena, Mª Victoria Fernández, Marta Marquié, Sergi Valero, Xavier Montalbán, Antonio Camins, Alfredo Ramírez, Marcè Martí, Mª Isabel Pividori, Mercè Boada, Agustin Ruiz, Miren Ettcheto, Amanda Cano

**Affiliations:** Research Center and Memory Clinic. Ace Alzheimer Center Barcelona; Biosensing and Bioanalysis Group, Institute of Biotechnology and Biomedicine, Universitat Autònoma de Barcelona; Research Center and Memory Clinic. Ace Alzheimer Center Barcelona; Research Center and Memory Clinic. Ace Alzheimer Center Barcelona; Department of Pharmacology, Toxicology and Therapeutic Chemistry, Faculty of Pharmacy and Food Science, Universitat de Barcelona; Department of Pharmacology, Toxicology and Therapeutic Chemistry, Faculty of Pharmacy and Food Science, Universitat de Barcelona; Research Center and Memory Clinic. Ace Alzheimer Center Barcelona; Research Center and Memory Clinic. Ace Alzheimer Center Barcelona; Research Center and Memory Clinic. Ace Alzheimer Center Barcelona; Research Center and Memory Clinic. Ace Alzheimer Center Barcelona; Research Center and Memory Clinic. Ace Alzheimer Center Barcelona; Research Center and Memory Clinic. Ace Alzheimer Center Barcelona; Research Center and Memory Clinic. Ace Alzheimer Center Barcelona; Research Center and Memory Clinic. Ace Alzheimer Center Barcelona; Research Center and Memory Clinic. Ace Alzheimer Center Barcelona; Research Center and Memory Clinic. Ace Alzheimer Center Barcelona; Research Center and Memory Clinic. Ace Alzheimer Center Barcelona; Research Center and Memory Clinic. Ace Alzheimer Center Barcelona; Research Center and Memory Clinic. Ace Alzheimer Center Barcelona; Research Center and Memory Clinic. Ace Alzheimer Center Barcelona; Research Center and Memory Clinic. Ace Alzheimer Center Barcelona; Research Center and Memory Clinic. Ace Alzheimer Center Barcelona; Research Center and Memory Clinic. Ace Alzheimer Center Barcelona; Department of Pharmacology, Toxicology and Therapeutic Chemistry, Faculty of Pharmacy and Food Science, Universitat de Barcelona; Division of Neurogenetics and Molecular Psychiatry, Department of Psychiatry and Psychotherapy, Faculty of Medicine and University Hospital Cologne, University of Cologne; Grup de Sensors i Biosensors, Departament de Química, Universitat Autònoma de Barcelona; Biosensing and Bioanalysis Group, Institute of Biotechnology and Biomedicine, Universitat Autònoma de Barcelona; Research Center and Memory Clinic. Ace Alzheimer Center Barcelona; Research Center and Memory Clinic. Ace Alzheimer Center Barcelona; Department of Pharmacology, Toxicology and Therapeutic Chemistry, Faculty of Pharmacy and Food Science, Universitat de Barcelona; Research Center and Memory Clinic. Ace Alzheimer Center Barcelona

**Keywords:** plasma extracellular vesicles, Alzheimer’s disease, mild cognitive impairment proteomics, biomarkers

## Abstract

**Aims:**

Alzheimer’s disease (AD) is commonly diagnosed when neuronal damage is already established and irreversible. Achieving an accurate differential diagnosis in the preclinical and mild cognitive impairment (MCI) stage is one of the greatest challenges nowadays. Nanotechnological analysis of plasma extracellular vesicles (pEVs) are gaining attention as a promising tool for the early detection of AD pathology. This study aims to evaluate the proteomic profile of pEVs from patients with MCI and AD dementia to explore their potential as AD screening tools.

**Methods:**

pEVs were isolated by ultracentrifugation from 144 patients with MCI A-T-, MCI A+T+, and AD dementia. Nanoparticle tracking analysis and cryo-TEM were used to characterize the pEVs. CSF, serum and pEVs proteomics were carried out by using the multiplex PEA technology of Olink^®^ proteomics, Inflammation and Neurology Explore 384 panels (768 proteins).

**Results:**

Characterization results showed that isolated plasma fraction corresponded in shape, size and concentration to EVs. Many pEVs neurology proteins involved in AD pathology significantly correlated (r > ± 0.30, p < 0.05) with their CSF homonyms, but not with their serum’s. pEVs’ proteome correlated with common AD signatures (CSF Aβ42 and pTau181, plasma pTau181, MMSE, NBACE, and Qalb) showing similar patterns to those observed with CSF biomarkers. Several pEVs neurology proteins didn’t exhibit differences between the MCI A+T+ and AD dementia groups, whilst they did with MCI A-T-. Proteins in pEVs showed strong correlations with several measures of brain atrophy in MRI. Several neurology pEV proteins predicted conversion from MCI to AD dementia. Moreover, some of these showed a significant diagnostic accuracy of AD pathology.

**Conclusion:**

Preliminary results suggest that EVs biomarker signature could reflect AD pathology in the prodromal stages of AD continuum. However, further experiments are still needed for a better understanding of EVs’ role in AD development and pathology dissemination.

## 1. Introduction

Alzheimeŕs disease (AD), the main underlying cause of dementia, is responsible for 60–80% of the total number of dementia cases. In 2023, an estimated 416 million people globally were living with AD across the disease continuum, representing 22% of individuals over 50 years old [1]. According to the latest report of the *Global Burden of Disease Study* published in 2021, AD was one of the ten conditions with the highest age-standardized disability- adjusted life-years (DALYs) worldwide [2]. Moreover, AD is currently the only condition among the 10 principal mortality causes worldwide that is still lacks a preventative treatment or cure [3].

Although the exact etiology of AD remains unclear, its main pathological hallmarks are well established, such as the accumulation of senile plaques, composed of extra-neuronal deposits of amyloid-β peptide (Aβ), and neurofibrillary tangles, composed of intra-neuronal deposits of hyperphosphorylated tau (pTau) [4]. These molecular alterations start in the preclinical stages, when the clinical symptoms are not yet evident. The presence of AD pathology in the brain along with detectable cognitive alterations (that are not functionally limiting) is defined as the prodromal phase of AD, also described as mild cognitive impairment (MCI) due to AD. During this stage, the disease process reaches maximum levels of Aβ and pTau-mediated neuronal injury [5] and after that, neuronal damage is considered irreversible. However, not all patients with MCI have underlying AD pathology or progress to dementia. Therefore, identifying patients with elevated risk of AD dementia in the earliest stages is one of the greatest challenges in clinical practice [6, 7].

Current biological diagnostic methods, such as cerebrospinal fluid (CSF) biomarkers or positron emission tomography (PET) radiotracers, involve invasive and high-cost techniques. As a result, many efforts are being made to translate the research of new AD biomarkers to plasma [8–10]. However, since plasma remains presenting some disease-specificity issues in these patients, an increasing interest in plasma extracellular vesicles (pEVs) has emerged, as a novel source of biomarkers for the identification of many neurodegenerative diseases [11], especially in AD [12–14].

EVs are natural structures released by cells and surrounded by an invaginated phospholipid bilayer from the plasma membrane of the originating cell. These vesicles are secreted by most body cells, and have been described to be involved in many physiological functions, like cellular communication, immune response, or metabolic processes [15, 16]. Moreover, due to their lipid composition, EVs can reach other organs, or even cross the blood brain barrier (BBB) [17], giving them unique potential as a source of brain-derived biomarkers at peripheral level.

Multiple studies support the hypothesis that highlight the role of EVs in AD pathogenesis. Although their exact function is not yet well known, it has been described that Aβ and pTau could be released in association with EVs, thus influencing neuronal cell death and transsynaptic spreading of the disease [18, 19]. Likewise, AD brain-derived EVs have also been shown to spread tau pathology in interneurons [20]. A recent study revealed that plasma EVs isolated from patients with AD, MCI, and healthy cognitive status showed an enriched mRNA AD pathway in those patients with the pathology and a strong correlation to the established Clinical Dementia Rating (CDR) [21]. All these evidences highlight the importance of investigating the EVs molecular content to understand their involvement in AD development.

In this context, the aim of this study was to evaluate the proteomic profile of pEVs from patients from a memory clinic to explore their potential as AD screening tools. To do that, we selected a group of 144 patients with MCI due to AD, MCI not due to AD, and AD dementia, and performed a massive screening of the proteomic signature of their isolated pEVs using the Inflammation and Neurology panels of Olink^®^ Explore technology.

## 2. Methods

### 2.1 Standard protocol approvals, registrations and patient consents

All study protocols were approved by the Clinical Research Ethics Commission of the Hospital Clinic (Barcelona, Spain, reference num: HCB/2014/0494) and Hospital Universitari de Bellvitge (Barcelona, Spain, reference num: PR152/23) in accordance with the Declaration of Helsinki and the current Spanish regulations in the field of biomedical research (law 14/2007, royal decree 1716/2011). Likewise, in accordance with Spain’s Data Protection Law (organic law 3/2018), all participants were informed about the study’s goals and procedures by a neurologist before signing an informed consent form. Patient privacy and data confidentiality were protected in accordance with applicable laws.

### 2.2 Participants, study groups, and selection criteria

All paired CSF/plasma samples were collected and registered in the Instituto de Salud Carlos III with the code C.0000299. A total of 144 patients were included in the study and divided into three groups: (i) patients with MCI and A + T + N(+/−) status in CSF [MCI A + T+, n = 50], (ii) patients with MCI and A-T-N(+/−) status in CSF [MCI A-T-, n = 50], and (iii) patients with AD dementia and A + T + N(+/−) status in CSF [AD dementia, n = 44]. All participants included were real-world patients evaluated and diagnosed at the memory clinic of the Ace Alzheimer Center Barcelona (ACE), (Barcelona, Spain). Briefly, a consensus diagnosis was assigned to each patient by a multidisciplinary team of neurologists, neuropsychologists, and social workers. Voluntary (with informed consent) lumbar punctures were offered to individuals with MCI and dementia who were evaluated at the Memory Clinic of ACE [22]. All clinical diagnosis consisted of a primary, secondary, and a syndromic diagnosis, accompanied by a biological diagnosis with CSF biomarkers and the AT(N) classification at the baseline visit [22, 23]. All patients with MCI were followed up annually to record their clinical progression. A range of 0.5 to 7 years of clinical follow-up (FU) data were included in this study.

### 2.3 Brain MRI images

Patients with AD dementia underwent a brain MRI within a period of less than 3 months from the lumbar puncture with a Siemens VIDA 2T at Clínica Corachán’s Radiology Department, (Barcelona, Spain) as described elsewhere [24]. MRI studies were examined by a group of experienced neuroradiologists and reported according to standard practice. The images were processed at ACE’s Neuroimaging Laboratory with Free surfer 6.0.1 (https://surfer.nmr.mgh.harvard.edu/). All brain areas were normalized by brain total volume to perform the analysis.

### 2.4 CSF and Plasma sample collection

CSF samples were used to measure common AD biomarkers and obtain the AT(N) classification. Plasma samples were used to isolate the EVs. Plasma and CSF samples were collected on the same day from each patient as described elsewhere [25, 26]. Briefly, blood samples were collected in polypropylene tubes with EDTA (BD Vacutainer). Plasma was separated by centrifugation (2000xg, 10 min, 4°C), aliquoted, and stored at −80°C until use. CSF was obtained by lumbar puncture (LP). An expert neurologist at Ace Alzheimer Center Barcelona performed LP in accordance with established consensus recommendations [27]. The patient was fasted, placed in a sitting position, and anesthetized with 1% subcutaneous mepivacaine. 13 mL of CSF were collected in polypropylene tubes (Sarstedt Ref 62.610.018). CSF was centrifuged for common AD biomarker determination (2000xg, 10 min, 4°C), and the supernatant was aliquoted and stored at −80°C until use. The collection protocol followed the recommendations of the *Alzheimer’s Biomarkers Standardization Initiative* [28].

### 2.5 DNA extraction, APOE genotyping and PRS determination

DNA extraction from blood specimens was performed automatically using standard procedures with the DNA Chemagic system (Perkin Elmer) or the Maxwell RSC48 instrument (Promega). Extensive DNA quality control was conducted, and only samples with DNA concentrations greater than 10 ng/μl and high integrity were included for *APOE* genotyping. The isoforms were determined by TaqMan probe analysis in the Real-Time PCR QuantStudio3 (ThermoFisher, Waltham, Massachusetts, USA) system or extracted from Affymetrix Axiom SP biobank arrays processed as previously described [29, 30]. AD PRS was computed as described in Bellenguez *et al*, which considered 83 loci [31]. For polygenic risk score (PRS) calculation, we added the gene dosages of single nucleotide polymorphisms that showed a high imputation quality, by weighting their effect size (beta coefficients); the allele analyzed was matched to the reported allele (A1) by Bellenguez *et al* [31]. For PRS computation, the effect (beta coefficients) and standard errors were estimated using the equations described by Zhu *et al* [32].

### 2.6 EVs isolation and characterization

The gold standard ultracentrifugation (UC) method was used to isolate and purify pEVs from plasma samples as previously described [26]. Briefly, 3 mL of plasma samples were centrifuged (10,000 g, 30 min, 4°C) to remove cellular debris, and the supernatant was ultracentrifuged twice (100,000 g, 60 min, 4°C) to remove microvesicles and other debris, and then to pellet the pEVs. All centrifugations were done with a Sorvall Discovery M150 SE (Thermo Scientific) Ultracentrifuge using an S100AT6 rotor. Nanoparticle tracking analysis (NTA), using a NanoSight LM10-HS system with a tuned 405 nm laser (NanoSight Ltd., UK), was used to analyze the concentration and particle size distribution of the pEVs. Cryogenic transmission electron microscopy (cryo-TEM) was used to determine the morphology of the pEVs. Images were collected by a Jeol JEM 2011 (JEOL USA Inc., USA) using an accelerating voltage of 200 kV. The total protein concentration of the obtained pEVs was measured using the Pierce^™^ BCA Protein Assay Kit (Thermo Fisher).

### 2.7 AT(N) classification and pEVs proteomics

For the AT(N) classification, on the day of the analysis, a CSF aliquot was thawed and Aβ_1−40_, Aβ_1−42_, t-Tau, and pTau181 biomarkers were quantified by using the Lumipulse G 600 II automatic platform (Fujirebio Inc.) as described elsewhere [25, 33]. In addition, plasma pTau181 was measured with the Lumipulse G1200 automatic platform (Fujirebio Inc.).

Proteomics of pEVs were conducted by using the multiplex Proximity Extension Assay (PEA) technology developed by Olink^®^ Proteomics (Uppsala, Sweden) as described elsewhere [34], and following the manufacturer’s protocol [35]. Briefly, pEVs were previously lysated and 1 μl were used to analyze two different commercially available panels: Olink^®^ Explore 384 *Inflammation* I panel and Olink^®^ Explore 384 *Neurology* I panel, both together allowing the detection of 768 proteins simultaneously. In addition, 1 μl of CSF and serum samples from both MCI groups were also measured with the Olink^®^ Explore 3072 panel. Protein levels were expressed as normalized protein expression (NPX) values on the log_2_ scale.

### 2.8 Principal component analysis

Principal Component Analysis (PCA) was conducted to evaluate the variation captured across the included proteins which overcame the QC. Proteomic plasma data was decomposed into multiple orthogonal principal components (PCs) and the proportion of variance explained by each component, as well as the cumulative variance, was calculated for each dataset. To assess unadjusted associations between PCs and clinical variables, Pearson correlation analyses were conducted between the top 5 PCs and the most relevant clinical variables (Age, sex, BMI, CSF Aβ42, ptau181 and total protein levels, QAlb, APOE genotype, AT(N) status, syndromic diagnosis, plasma glucose, dyslipidemia and sample storage duration at −80°C). To further dissect the independent sources of variance, multivariable linear models were fitted for each of the top five PCs including all clinical variables simultaneously.

### 2.9 Enrichment analysis

PANTHER classification system version v17.071 was used to elucidate the subfamilies and functions of the measured proteins and annotate them by using a Homo sapiens reference gene list. A statistical overrepresentation test was performed to associate these proteins with PANTHER GO-Slim terms such as Cellular Compartment, Biological Process and Molecular Function using Fisher's exact test. In addition, functional classification was performed to annotate the protein class of each analyte.

### 2.10 Statistical analysis

All statistical analyses were conducted using R studio version 4.2.2, SPSS, and GraphPad Prism 8.0. Risk stratification of the subjects in each study group was performed using the A/T/(N) classification as described elsewhere [25]. Data were log-normalized and Z-score standardized. NPX values were Z-score standardized and used as input for the data analysis pipeline. In the quality control (QC), values below the limit of detection (LOD) and proteins with over 30% missing values were excluded from the analysis. A One-way ANOVA followed by Tukey's post hoc test was used to compare the different protein levels across groups. Pearson correlations and simple linear regression, which included a general least squares method, were used to evaluate the associations between CSF, serum and pEVs’ protein levels. Multivariable linear models correlation analysis were used for PCs analysis. Receiver operating characteristic (ROC) curves were plotted by using the *roc* and *coords* functions from the R package *pROC* to evaluate the test performance of identified significant proteins. Sensitivity minimum threshold was set at 90.0% and the highest specificity were fixed to empower the findings. *Kaplan-Meier* curves were used to evaluate the ability of pEVs to predict the risk of conversion to dementia in the MCI group. The start point was the baseline visit (when CSF was collected and the initial diagnosis was made), and end points were i) conversion to AD dementia for converters and ii) last follow-up visit for non-converters.

## 3. Results

### 3.1 pEVs Characterization

**Figure 1:**
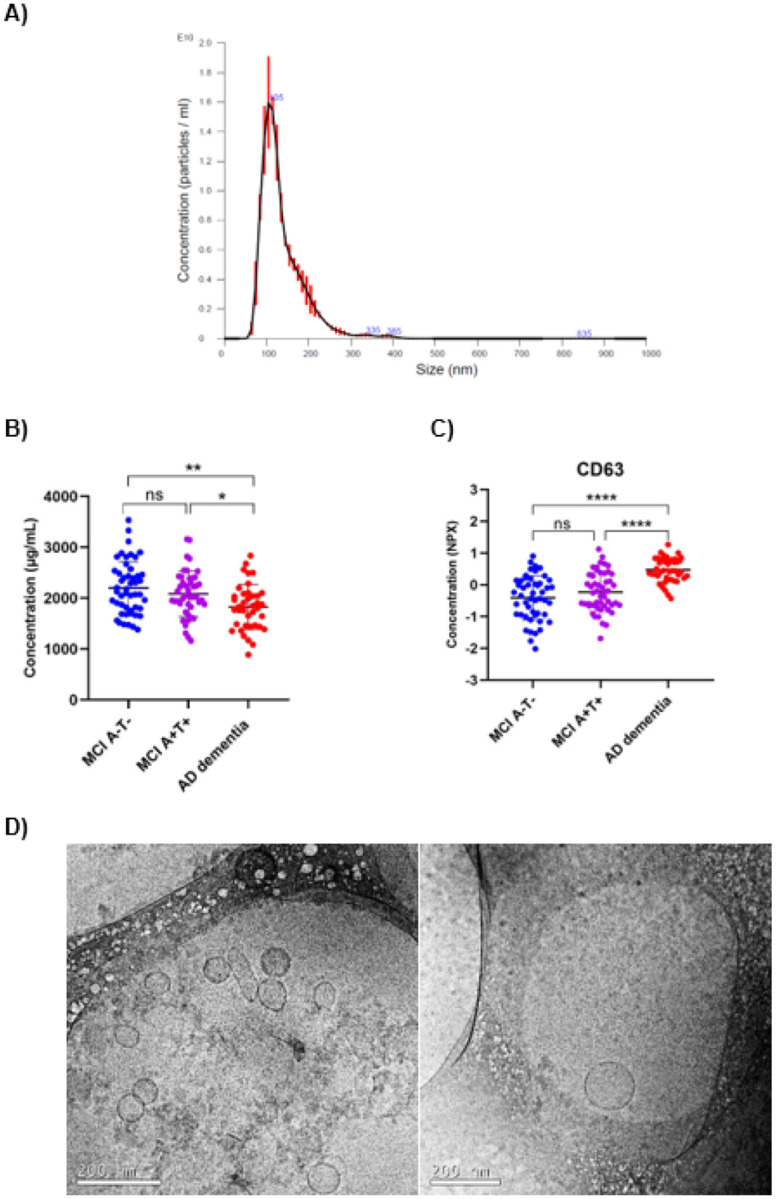
Characterization results of isolated pEVs. **A)** Averaged FTLA Concentration / Size of pEVs samples measured by NTA analysis. Measurements were run by triplicate. **B)** Histogram shows the Pierce BCA total protein results of isolated pEVs. Statistical analysis was performed with a one-way ANOVA analysis with a post-hoc Tukey's multiple comparisons test. **C)** Histogram shows the comparison of CD63 protein in the isolated pEVs between the three study groups. Statistical analysis was performed with a one-way ANOVA analysis with a post-hoc Tukey's multiple comparisons test. **D)**Representative cryoTEM images of isolated pEVs. Scale bar 200 nm. Statistical analysis was performed with a one-way ANOVA analysis with a post-hoc Tukey's multiple comparisons test. p < 0.05 *, < 0.01 **, <0.001 ***, <0.0001 ****.

Characterization results showed that isolated plasma fraction corresponded in shape (smooth spherical), average size (113.4 ± 4.7 nm) and average concentration (1.22e11 ± 2.50e09 particles/ml) to EVs. Total protein content analysis of pEVs revealed that AD dementia group showed a statistically significant reduction in the total protein concentration compared to both MCI groups, which did not show any difference between them. In contrast, CD63 levels, a tetraspanin included in the Neurology panel and often used as a specific biomarker for EVs [36], were significantly elevated in the AD dementia group, suggesting an increased production of pEVs in pathological conditions (Fig. 1).

### 3.2 Demographics, missingness and protein selection

Patients included in the study exhibited the common characteristics widely described for MCI and AD dementia. In this sense, AD dementia group was composed of a higher proportion of females compared to both MCI groups(which had a similar sex distribution) [37], an older population, and lower years of education (Table 1). BMI, as well as CSF and blood biochemistry were similar among study groups. The AD dementia and MCI A + T + groups showed similar values in the common CSF and plasma AD biomarkers. As expected, AD dementia group had the lowest scores on the MMSE and NBACE tests, increased proportion of ApoE4 alleles (59.1% compared to 16.0% and 52.0% in MCI A-T- and MCI A + T + groups respectively). PRS appeared to be similar among the study groups. Regarding protein selection, out of the 768 proteins analyzed with Olink Explore, 662 overcame the QC (335 and 327 from the Neurology I and Inflammation I panels respectively) (Table 1).

### 3.3 Protein levels among study groups

**Figure 2:**
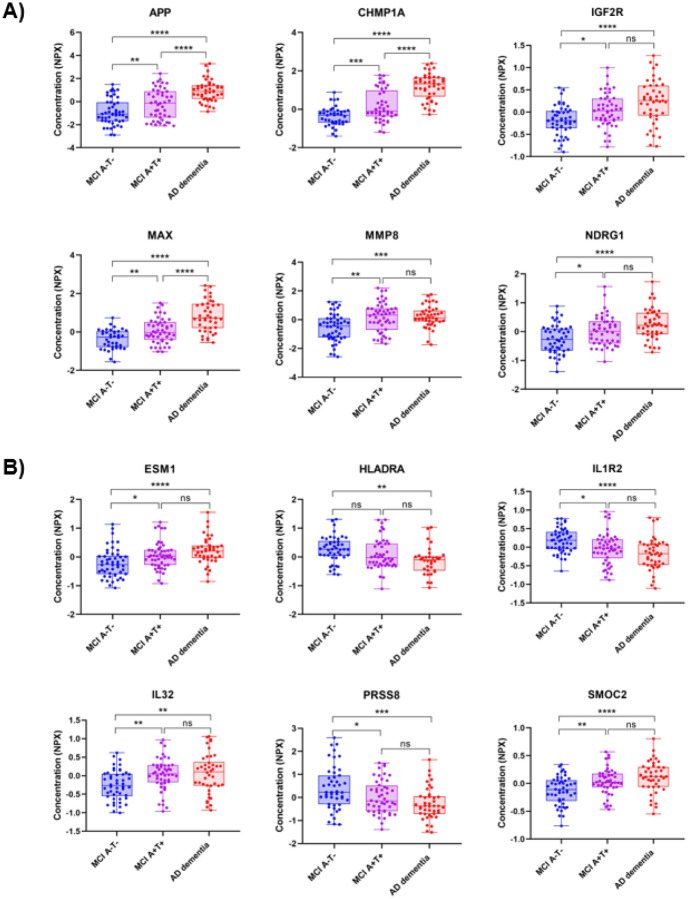
Histograms show the comparison of protein levels among the three study groups of top six most relevant proteins of **A)** Neurology pathway and **B)** Inflammation pathway. Statistical analysis was performed with a one-way ANOVA analysis with a post-hoc Tukey's multiple comparisons test. p < 0.05 *, < 0.01 **, <0.001 ***, <0.0001 ****.

The analysis of the protein levels among the study groups revealed that 62.0% of the proteins included in the study showed significant differences between the AD dementia and the MCI groups in the pEVs fraction, with the vast majority being increased (59.5% of the total proteins, 96.0% of the significant ones) (Table 1). Moreover, 20.5% of the proteins that passed the QC showed significant different levels in the EVs compartment between the MCI A-T- and MCI A + T + groups. APP appeared to increase in the pEVs as the disease progressed along the AD continuum (Fig. 2). Interestingly, a small group of proteins presented significant differences between the AD dementia and the MCI A−T− group, but not with the MCI A + T + group (3.6%), suggesting a specific AD related pathway of these molecules in the pEVs (Fig. 2). Detailed data are shown in **Table S1** of Supplementary material.

### 3.4 Correlation of pEVs proteins with main AD hallmarks and clinical variables

**Figure 3:**
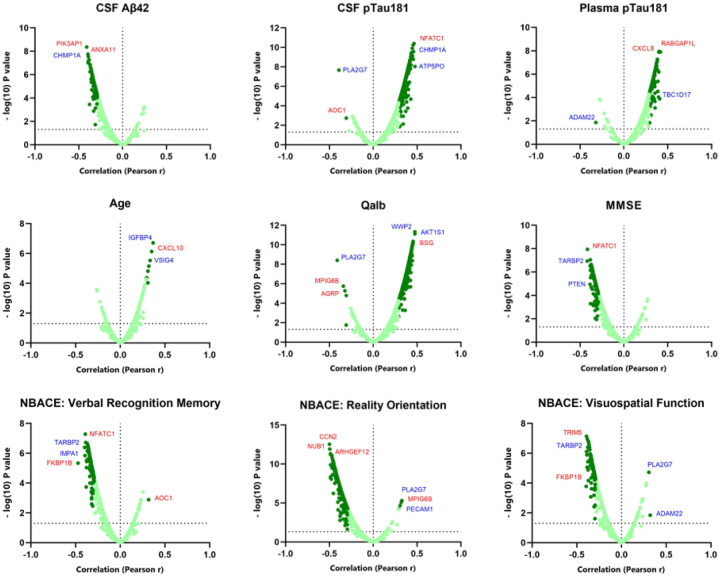
Volcano plots of the associations between pEVs protein levels and main AD clinical variables. Graph is expressed by the magnitude of correlation (−log(10) p Value) *vs* fold-change, expressed by Pearson r, of the correlation. Dark dots refer to proteins with Pearson r > 0.30. Horizontal grid line represents the significant p Value threshold (p<0.05). Proteins labelled in blue belong to the Neurology panel. Proteins labelled in red belong to the Inflammation panel.

The association study of proteins with main AD clinical features showed that pEVs fraction reproduced the pattern previously reported in CSF [38–40]. In this sense, pEVs proteins showed a strong negative correlation with CSF Aβ42 and a positive correlation with both CSF and plasma pTau181, with many proteins showing statistically significant associations. A similar result was observed for Qalb. Age and ApoE4 presence showed a slight tendency towards a positive correlation, although few proteins reached statistical significance. Sex, BMI, PRS, years of education, blood and CSF glucose levels, and total globulins in both CSF and serum did not show this polarized distribution or a wide proportion of strong correlations. Interestingly, pEVs proteins exhibited a strong negative correlation with several cognitive tests, including the MMSE and different domains of the NBACE battery (Fig. 3, **Table S2**).

Notably, MMP8 showed a strong correlation with ApoE4 (r = 0.38, p < 0.0001) and FCRL5 with total globulins in serum (r = 0.60, p < 0.0001), both proteins from the Neurology panel; while in the Inflammation panel, PSPN was associated with sex (r=−0.47, p < 0.0001) and CXCL10 with age (r = 0.37, p < 0.0001. Likewise, FKBP1B and TARBP2 (from the Inflammation and Neurology panels respectively), both involved in immune system processes and cell signaling, were strongly negatively correlated with many cognitive and neuropsychological scores (**Table S2**).

When these parameters were analyzed in serum, only age and plasma pTau181 maintained a similar degree of correlation, whilst all the other parameters lost statistical significance as well as correlation strength (**Figure S2**).

### 3.5 Enrichment and Principal Component analysis

**Figure 4:**
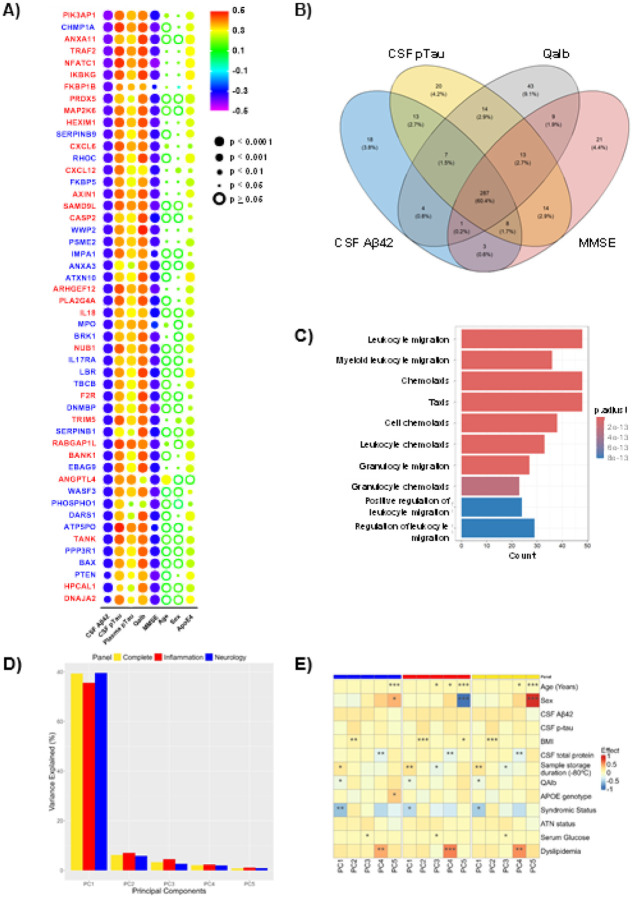
**A)** Top 50 pEVs proteins highly correlated with CSF Aβ42 levels. The color scale represents the degree of correlation (Pearson r) and the size of the dots the statistical significance (p value). Proteins labelled in red and blue belongs to the inflammation and neurology panels respectively. **B)** Venn diagram of CSF Aβ42, CSF pTau, Qalb and MMSE significant associations with complete set of Olink Explore pEVs proteins (n=662). **C)**Top ten identified enriched molecular pathways in pEVs protein using the Panther tool. Statistical significance was set at p value < 0.05. **D)** Variance explained (%) by PCs 1–5. **E)** Multiple linear regression correlation analysis. Variables included in the model: demographic, clinical, and CSF biomarkers.

When analyzing the uniquely significant pEVs proteins (p < 0.05) associated with main AD parameters, it was found that 51.5% (n = 341) of proteins that passed QC were significantly associated with Aβ42 levels in CSF, 56.8% (n = 378) with pTau levels in CSF, 57.1% with Qalb, and 53.8% with MMSE. Among all the pEVs proteins associated with at least one of these parameters (n = 475), 60.4% (n = 287) were significantly associated with all four AD features, followed by 9.1% (n = 43) associated exclusively with Qalb and 4.4% (n = 21) associated exclusively with cognition (Fig. 4A and 4B, Table 2). Enrichment analysis showed that the immune system processes, mainly immune cell migration and chemotaxis were the most prominent molecular pathways across all associations (Fig. 4C). However, the targeted nature of the protein panels used in these analyses inherently affects this finding.

PC analysis revealed that the first PC (PC1) accounted for the vast majority of variance across all proteins (Fig. 1D, complete panels: 79.3%; Inflammation: 75.5%; and Neurology: 79.6%), indicating a strong dominant axis of variation shared across panels. The cumulative variance explained by the first 20 components exceeded 95% for all panels (**Figure S3**), suggesting a relatively low-dimensional structure within the datasets. Multivariable linear models correlation analysis showed that CSF total protein, age, QAlb and sample storage duration at − 80°C were consistent, independent contributors to variance across panels (Fig. 1E). In contrast, variables such as CSF Aβ42 and p-Tau181, plasma glucose or APOE genotype exhibited few or weak associations across all PCs, suggesting a limited influence on the primary axes of proteomic variability after accounting for covariate interactions. Interestingly, despite panel-specific differences in protein targets, the dominant components of variance remained largely similar, with PC1 capturing shared biological signals tied to CSF biomarker levels and sample handling.

### 3.5 Associations of pEV proteins with brain volumes and cerebrovascular pathology

**Figure 5:**
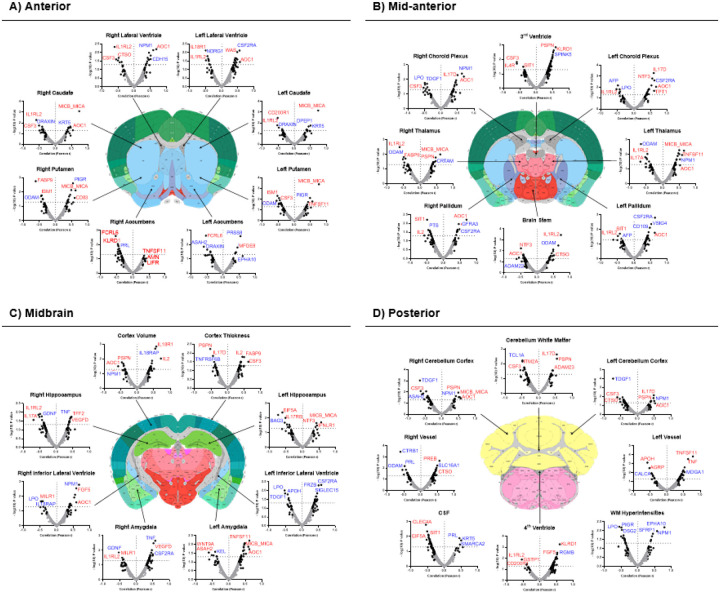
Associations of pEVs proteins with volumes of different brain areas defined by MRI, distributed in **A)** anterior brain, **B)**middle anterior brain, **C)** midbrain, and **D)** posterior brain. Brain atlas images were used as a schematic representation of brain distribution and obtained from http://atlas.brain-map.org/. Volcano plots show the associations between pEVs protein levels and main MRI brain region volumes. Plots display correlation strength (−log(10) p Value) *vs* fold-change, represented by Pearson correlation coefficient (r).. Dark dots refer to proteins with Pearson r > 0.30. Horizontal grid line marks the significance p Value threshold (p<0.05). Proteins labelled in blue belong to the Neurology panel. Proteins labelled in red belong to the Inflammation panel.

pEVs proteins from both panels were associated with MRI data to evaluate the relationship between the proteomic signature of pEVs and the volumes of different brain regions in confirmed AD cases. The obtained results showed that pEVs proteins exhibited a positive correlation with several brain ventricles, especially with the 3rd ventricle (Fig. 5B). Most of the associated proteins belonged to the inflammatory panel. In contrast, volumes of several structures such as the *nucleus accumbens* or the CSF volume appeared to be negatively correlated (Fig. 5A, 5D). In addition, all pEVs proteins strongly associated with white matter (WM) hyperintensities were from the Neurology panel (Fig. 5D). Interestingly, several proteins such as MICB_MICA, AOC1, PSPN or NPM1 were strongly associated with volumes of key brain regions implicated in AD, including the *hippocampus*, brain cortex or the *amygdala* (Fig. 5C).

### 3.6 Correlation of pEV proteins with both CSF and serum biomarkers

**Figure 6:**
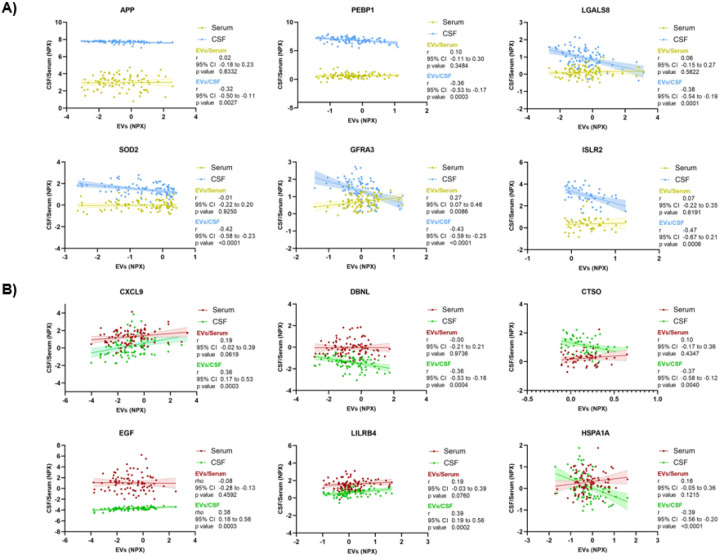
Top six pEVs protein correlations with their CSF and serum homologues of A) Olink^®^ Explore Neurology I panel and B) Olink^®^ Explore Inflammation I panel.

Correlation analysis between pEVs proteins and their homologues in both CSF and serum showed that main significant results (r > ± 0.3, p < 0.05) appeared to be positively correlated with serum and negatively correlated with CSF in both panels (**Figure S4**). Interestingly, a substantial group of proteins was significantly correlated with CSF but not with serum, suggesting that pEVs may reflect a peripheral signature of central nervous system (CNS) proteomics (Fig. 6). In addition, some of these proteins highly correlated with CSF, such as APP, PEBP1, LGALS8, SOD2 or ISLR2 have been described to be specifically involved in AD molecular pathology [41–45]. Likewise, several pEVs inflammation proteins known to be increased in patients with AD, such as CXCL9 [46], were strongly positively correlated with CSF but not with serum. These findings indicate the specificity of pEVs compared to whole blood, as a peripheral mirror of the CNS proteomic signature in AD.

### 3.7 pEV proteins as predictors of conversion to AD dementia

**Figure 7:**
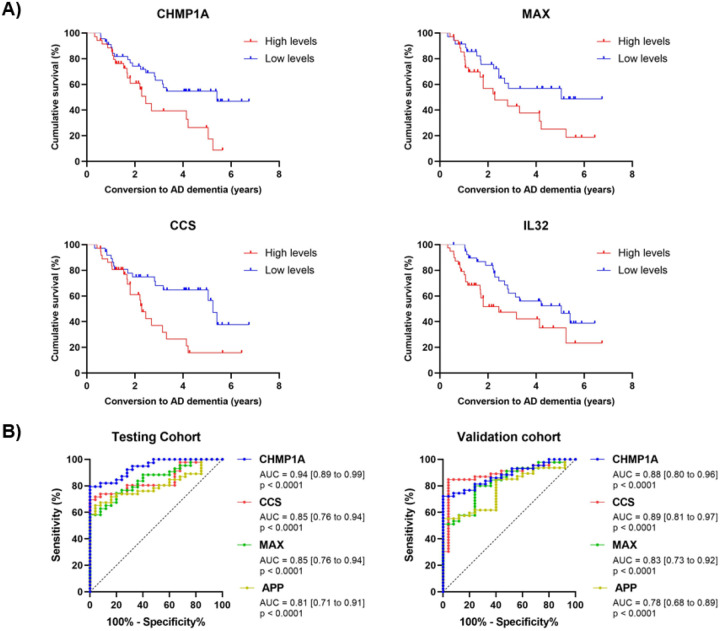
**A)** Survival curves from *Kaplan-Meier* analysis of pEV proteins that significantly predicted the MCI conversion to AD dementia. “Cumulative survival” refers to the time until conversion to AD dementia. **B)** ROC curves of identified significant proteins in the pEVs fraction. Patients with negative and positive AD pathology (A−T− / A+T+) were selected as “Controls” and “Cases” respectively. All proteins belong to the neurology panel.

Proteins from pEVs were analyzed to assess their ability to predict progression to AD in the MCI subgroups. For each protein, MCI individuals were divided in two groups: i) patients with MCI and FU data with high levels of proteins (medium to maximum detected values) and ii) patients with MCI and FU data with low levels of proteins (medium to minimum detected values). Cumulative survival was defined as the time until conversion to AD dementia. Survival curve comparison showed that four proteins significantly predicted conversion to AD dementia (Fig. 7A). Three of these proteins, CHMP1A, MAX and CCS, were from the Neurology panel and IL32 from the Inflammation panel. As shown in Table 3, CCS, previously described to impact APP processing and increase Aβ production in AD [47], exhibited the strongest predictive value (X^2^ = 6.72, p = 0.0095). Notably, patients with high levels of these proteins in pEVs had a median survival time ranging from 2.3 to 2.4 years, whereas patients with low levels doubled this value, ranging from 5.0 to 5.4 median survival years. Moreover, high CCS levels in pEVs showed a hazard ratio (HR) of 2.5 for increased risk of conversion to AD dementia. Similar results were observed for the other three identified proteins.

When this analysis was repeated using the CSF and proteomic dataset, none of the four proteins passed QC. In the serum dataset, only CCS and IL32 passed QC, but neither of them showed significant results in the survival analysis (CCS X^2^ = 0.09, p = 0.7567; IL32 X^2^ = 1.91, p = 0.1672) (**Figure S3, Table S3**).

### 3.8 Diagnostic performance of pEV proteins to predict AD pathology

Different proteins were studied to examine the diagnostic potential of pEVs cargo thought the ROC curve analysis. Patients were randomly divided in two cohorts [testing cohort (n = 71) and validation cohort (n = 72)] to perform the analysis.

Four proteins from the neurology panel (CHMP1A, CCS, MAX and APP) exhibited AUCs > 0.80 and statistical significance in the testing cohort (Fig. 7B). CHMP1A levels in pEVs showed the best diagnostic accuracy of AD pathology (AUC = 0.94 [0.89 to 0.99], p < 0.0001), with sensitivity of 89.7% [77.4 to 96.9], specificity of 72.0% [52.2 to 85.7], positive predictive value (PPV) of 83.3% [67.3 to 91.0], negative predictive value (NPV) of 81.8% [66.2 to 90.4], and Youden Index of 61.7. In the scenario of a memory clinic, this diagnostic accuracy would save 34.4% of performed lumbar punctures in these group of patients. At predefined value of sensitivity close to 90.0%, none of the other three identified proteins showed a specificity ≥ 70.0%. Validation cohort exhibited similar results, corroborating these findings (Table 4).

## 4. Discussion

A new case of dementia is diagnosed every 3 seconds worldwide [48]. In the absence of a cure, and with the expected rise in incidence and consequently, the economic and social burden of the AD dementia epidemic, an early, precise and affordable diagnosis is crucial to avoid threatening the sustainability of healthcare systems around the world. Nanomedicine and nanotechnology are revolutionizing the screening and diagnosis of patients of those diseases with complex etiology and multifactorial molecular pathways. In this study, we explored the potential of pEVs as a peripheral source of biomarkers for the detection of AD pathology in prodromal stages. To this end, we isolated the pEVs of patients with MCI (both positive and negative AT status) and AD dementia, and measured the levels of 768 proteins related to neurological, immune, and inflammatory processes.

Characterization results showed that the isolated fraction matched the expected shape, size and concentration of EVs, consistent with findings reported in other studies [49, 50]. The total protein content of pEVs was also reduced in patients with AD dementia as previously described [26]. However, CD63 levels, one of the most common tetraspanins found in EVs (particularly exosomes) [15], were significantly higher in the AD dementia group, suggesting increased pEV biogenesis and exosome enrichment. In pathological conditions, overproduction of exosomes has been described [15, 51]. This process is hypothesized to result from an increased need for cellular communication, the activation of immune and non-immune processes, or the propagation of disease hallmarks [15], which aligns with pathophysiological mechanisms observed in AD.

When comparing the levels of the included proteins across the three study groups, 20.5% showed significant differences between the diseased groups (MCI A + T + and AD dementia) and the non-neurodegenerative group (MCI A-T-). pEVs APP levels appeared to increase progressively as the disease advanced along the AD continuum. Although APP is not exactly equivalent to Aβ42, this finding could be related to those found by Winston *et al*., who reported that concentrations of Aβ42 were significantly increased in pEVs of neuronal origin in patients with MCI [52]. Similarly, proteins such as MMP8, IL32, NDRG1, SMOC2, or ESM1, which have been previously reported to contribute to AD pathology [53–57], showed to be significantly increased in both MCI A + T + and AD dementia, but not in MCI A-T-, suggesting a specific AD-related signature in pEVs proteome. Interestingly, proteins such as HLADRA, which plays a role in suppressing Tcell responses [58], or PRSS8 and IL1R2, both of which have anti-inflammatory effects [59, 60], appeared to be reduced in the diseased groups, further supporting the hypothesis of an AD-specific signature in the pEVs.

The association study with main AD biological and clinical features showed that increased levels of pEVs proteins strongly correlated with reduced levels of soluble Aβ42 in CSF, a typical pattern found in AD and a key diagnostic biomarker [25]. Regarding pTau, the other primary AD biomarker, the analysis revealed that pEVs proteome was also strongly correlated, in this case positively, with increased levels of both CSF and plasma pTau181. CHMP1A, from the Neurology panel, was among the most strongly correlated proteins with both CSF Aβ42 and pTau (r = 0.39, p < 0.0001 and r = 0.46, p < 0.0001 respectively). This protein is involved in multivesicular body sorting of cargo proteins to lysosomes, a pathway commonly associated with EV clearance [15]. Interestingly, in a recent study by Hondius *et al*., CHMP1A was found to be increased in neurons with granulovacuolar degeneration, a common feature in AD closely associated with NFTs [61]. Another parameter that showed strong correlation with increased protein levels in pEVs was Qalb. Since an elevated Qalb value is indicative of BBB damage, one of the main alterations occurring in AD, it is plausible that increased BBB permeability facilitates the crossing of brain-derived EVs into the bloodstream. Supporting this, numerous proteins from the Neurology panel were significantly correlated with Qalb includingAKT1S1 (r = 0.48, p < 0.0001), WWP2 (r = 0.47, p < 0.0001), LBR (r = 0.45, p < 0.0001), and RHOC (r = 0.44, p < 0.0001), all of which have also been reported to be implicated in Aβ and tau accumulation, and AD development [62–65]. pEVs CHMP1A was also strongly associated to Qalb (r = 0.44, p < 0.0001). Another relevant finding was the strong association between MMP8 and ApoE4 presence, as well as the significant negative correlation of pEVs proteome and MMSE scores, along with several neuropsychological domains, such as *reality orientation or verbal recognition memory*. These domains contribute to episodic memory processing, especially hippocampal-dependent functions, which are well known to be impaired in AD [66].

The enrichment analysis results were aligned with the findings described above, highlighting the involvement of immune system pathways and cytokine chemotaxis in pEVs proteome. Neuroinflammation cascades and immune system processes are widely recognized as key contributors to the development of AD [67]. In addition, most of the pEVs proteins significantly correlated with main AD features (CSF Aβ42 and pTau, Qalb and MMSE) were involved in Akt signaling pathways (widely described to be one of the main molecular routes in AD), as well as, inducible expression of cytokine genes in T-cells, NF-kappa-B activation, intracellular trafficking, and multivesicular bodies formation (the initial step of EV biogenesis). Altogether, these results reinforce the key role of pEVs in AD-related pathways. Related to that, one of the most interesting findings of this study was the correlation between many neurology pEVs proteins with their CSF homologues, but not instead with serums’. In this sense, we saw that the correlation patterns between pEV proteins and their homologues in CSF and serum were completely different between the two fluids, being mainly positive with serum (as expected, given that plasma was the origin source of EVs), and predominantly negative with CSF. Furthermore, a wide variety of pEVs proteins from both neurology and inflammation pathways showed stronger correlations with their CSF homologues than with those in serum. This finding highlights the purity of the isolated pEV fraction, reinforce the close connection between peripheral EVs and AD-brain molecular proteomic profile, and further supports the potential of pEVs as a peripheral screening tool for CNS-related diseases.

Regarding the PC analysis, results showed that CSF total protein, age, BBB disruption and sample storage time were the main independent contributors to the variance across all proteins. When evaluating the relationship between the pEVs proteome and the volumes of different brain regions, we found a clear positive correlation between the increase of pEVs levels and an enlargement of the 3rd and 4th ventricles, an effect closely related with brain atrophy processes in AD [68, 69]. This was in agreement with previous studies, reporting a positive correlation between pEVs inflammation proteins and increased 4th ventricle volume [26], as well as a link between elevated circulating inflammatory markers and greater ventricular volume [70]. Similarly, we found a strong correlation between increased pEVs protein levels and reduced CSF volume on MRI. Several MRI studies have shown that CSF flow is reduced at the clinical phase of AD and is associated with cognitive decline [71, 72], which is consistent with our findings. Regarding specific proteins, such MICB_MICA, known to activate natural killer cells, or AOC1, which regulates many biological processes including inflammation, appeared highly correlated with increased volumes of different brain regions typically affected in AD (i.e. *hippocampus* and *cortex*) as well as other areas, such as the *caudoputamen, thalamus* or the *amygdala*. While it could be expected that this correlation would be inverse, our findings suggest the possibility that some neuroinflammation-related molecules may help prevent plaque formation and its neurotoxic effects [73]. However, this mechanism is still not well understood. Similarly, increased volumes of these brain areas were also positively strongly correlated with some neurology biomarkers. One of these was NPM1, a protein implicated in other neurodegenerative diseases and known to interact with misfolded proteins, reducing their mobility and mitigating irreversible aggregation [74]. We hypothesize that NPM1 may be similarly interacting with Aβ aggregates in AD, potentially explaining the observed correlation: higher NPM1 levels could be associated with lower Aβ aggregation, reduced AD pathology and greater preservation of brain volume.

The other main finding of this study was the ability of some pEVs proteins to predict conversion from MCI to AD dementia. As shown in Fig. 6, neurology proteins such CHMP1A, MAX and CCS, and the inflammation protein IL32, were able to predict conversion with a difference of at least 2 years in median survival between the subjects with high levels and low levels of protein in the pEVs compartment. Interestingly, these proteins appeared also to be increased as long as the diseases progress when compared the protein levels between the study groups. CHMP1A levels in pEVs also showed the best diagnostic accuracy of AD pathology, with sensibility, specificity, PPV and NPV higher that 90.0%. This results were confirmed in a validation cohort. All these findings together significantly contribute to the hypothesis of the pEVs as peripheral mirrors of the CNS proteomic signature in AD.

Despite the great novelty and clinical relevance of our findings, this study has inevitable limitations that warrant consideration. First, the lack of healthy controls due to ethical and legal constraints on lumbar punctures restricted our ability to distinguish AD-specific changes from normal aging processes. While the study explores multi-compartment correlations, not all participants had matched MRI or CSF/serum proteomic data, which reduced statistical power in some analyses. Moreover, the inherent bias introduced by the limited and selective nature of the selected panels may overrepresent certain pathways and underrepresent others. However, pathway analyses and interpretation of the results were also performed separately for each panel to account for this issue. Finally, the high sample volume required to isolate the pEVs (3 ml per patient) resulted in a limited size cohort, which does not occur in plasma-based studies, which commonly use a few μL of sample, thus allowing to the performance of large patient cohorts’ assays. However, the results obtained are highly scientifically relevant and provide a strong foundation for future studies to validate these findings in independent cohorts.

In conclusion, our findings highlight the potential of pEVs as a source of biomarkers and their utility as peripheral nanosized screening tools for AD pathology in the earliest stages. However, further studies are needed to corroborate these findings and delve into the role of pEVs in the development of AD. With the emergence of the first disease-modifying treatments, requiring cutting-edge screening tools to select eligible patients, and the current paradigm of precision medicine, as recommended by the international clinical guidelines and reference institutions, it is highlighted the urgent need for new treatments and therapeutic targets. In this context, pEVs offer a promising alternative and represent a new strategy for screening, monitoring and diagnosing AD and other CNS pathologies.

## Supplementary Material

Supplementary Files

This is a list of supplementary files associated with this preprint. Click to download.

- Supplementarymaterialv4.pdf
- Table4.docx
- GA.png

## Figures and Tables

**Table 1:** Demographic data of the three study groups and selected proteins for comparative analysis

Demographic data	Study groups		
	MCI A−T−	MCI A+T+	AD dementia
	mean (SD)	mean (SD)	mean (SD)
**n**	50	50	44
**Female (%)**	46.0	44.0	59.1
**Age (years)**	68.8 (9.2)	74.4 (6.6)	75.3 (6.4)
**BMI**	26.8 (3.9)	26.6 (4.1)	25.5 (3.8)
**Years of Scholarship**	8.8 (4.6)	8.4 (4.7)	7.5 (4.8)
**Race/ethnicity**	NA	NA	NA
**CSF Aβ42 (pg/ml)**	1126.5 (255.8)	526.1 (136.8)	538.2 (128.4)
**CSF Aβ40 (ng/ml)**	12.9 (2.6)	12.4 (3.4)	12.6 (3.1)
**CSF pTau (pg/ml)**	41.0 (10.9)	104.5 (34.8)	136.0 (76.2)
**CSF tTau (pg/ml)**	268.0 (250.2)	669.4 (274.7)	781.3 (392.8)
**Plasma pTau181 (pg/ml)**	1.4 (0.7)	2.4 (0.7)	2.7 (1.0)
**Qalb**	0.9 (0.1)	0.9 (0.1)	1.0 (0.1)
**Glucose CSF (g/L)**	68.8 (22.3)	63.2 (8.9)	67.5 (11.5)
**Glucose blood (g/L)**	114.6 (37.0)	108.1 (18.8)	108.7 (20.5)
**Total globulines CSF (g/L)**	0.2 (0.1)	0.2 (0.1)	0.1 (0.1)
**Total globulines serum (g/L)**	27.6 (3.6)	27.5 (3.4)	27.2 (6.4)
**MMSE**	27.1 (2.5)	24.3 (3.1)	21.8 (3.6)
**NBACE reality orientation**	14.6 (0.9)	13.6 (1.6)	11.8 (2.5)
**NBACE visuospatial function**	3.2 (0.9)	2.5 (1.2)	2.0 (1.4)
**NBACE verbal recognition memory**	20.4 (2.7)	17.7 (3.2)	14.7 (4.0)
**Follow up data (n)**	38	43	NA
**Conversion to dementia (%)**	23.6%	69.8%	NA
**ApoE 2/2**	0.0	0.0	0.0
**ApoE 2/3**	14.0	4.0	2.3
**ApoE 2/4**	2.0	0.0	4.5
**ApoE 3/3**	70.0	40.0	34.1
**ApoE 3/4**	14.0	42.0	47.8
**ApoE 4/4**	0.0	10.0	6.8
**NA**	0.0	4.0	4.5
**AD PRS**	5.5 (0.4)	5.6 (0.3)	5.6 (0.3)
Proteins included in the study	Olink Explore panels		
	Neurology I	Inflammation I	Total
	n (%)	n (%)	n (%)
**Proteins that pass QC**	335 (87.2)	327 (85.2)	662 (86.2)
Proteins with significant differences between AD and both MCIs	161 (48.1)	193 (59.0)	354 (62.0)
Proteins with significant increased levels (AD dem > both MCIs)	156 (46.6)	184 (56.3)	340 (59.5)
Proteins with significant differences between MCI Ap(−) and MCI Ap(+)	57 (17.0)	79 (24.2)	136 (20.5)
Proteins with significant differences between AD dem and MCI Ap(−) but not with MCI AP(+)	7 (2.1)	17 (5.2)	24 (3.6)

**Table 2:** Top 10 correlated pEVs proteins with all main AD clinical features (CSF Aβ42 and pTau, Qalb and MMSE). Uniprot database was used for the identification of related molecular pathways and main protein functions.

	CSF Aβ42		CSF pTau		Qalb		MMSE		
Proteins	Pearson r	p Value	Pearson r	p Value	Pearson r	p Value	Pearson r	p Value	Main Functions and Involved Pathways
**Inflammation**									
**PIK3AP1**	−0.4097	4.36060E-09	0.4165	2.26763E-09	0.4001	1.06893E-08	−0.3212	9.28089E-06	Immune system process and Aktsignaling pathway.
**ANXA11**	−0.3946	1.76723E-08	0.3556	4.80091E-07	0.4523	5.73790E-11	−0.3560	7.56893E-07	Cytokinesis and vesicle-mediated transport
**TRAF2**	−0.3913	2.81723E-08	0.4547	5.57850E-11	0.4499	9.35100E-11	−0.3566	8.35860E-07	TNF receptor-associated factor
**NFATC1**	−0.3888	6.34062E-08	0.4654	4.06460E-11	0.4345	9.83609E-10	−0.4150	1.13279E-08	Inducible expression of cytokine genes in T-cells (IL-2 / IL-4)
**IKBKG**	−0.3821	5.32406E-08	0.4352	3.51487E-10	0.4544	4.55480E-11	−0.3637	4.18303E-07	NF-kappa-B essential modulator
**Neurology**									
**CHMP1A**	−0.3946	9.26599E-08	0.4591	2.6994E-10	0.4421	1.41996E-09	−0.3854	2.94307E-07	Multivesicular bodies (MVBs) formation, Cytoskeleton and membrane organization.
**SERPINB9**	−0.3697	1.51981E-07	0.394	1.86458E-08	0.4121	3.46368E-09	−0.3382	2.84071E-06	Immune system process, programmed cell death, molecular regulator activity.
**RHOC**	−0.3635	2.54262E-07	0.3938	1.90162E-08	0.4479	9.18400E-11	−0.3654	3.64869E-07	Regulates a signal transduction pathway linking plasma membrane and actin stress fibers. Serves as a microtubule-dependent signal, signaling and cell motility.
**FKBP5**	−0.3608	3.14668E-07	0.381	5.85987E-08	0.3769	8.35991E-08	−0.3555	7.88673E-07	Regulator of Akt pathway (activates Akt/AKT1), NF-kappa-Bactivation, and IFN production.
**Inflammation**									
									Plays a role in the intracellular trafficking.
**WWP2**	−0.3562	4.54996E-07	0.3851	4.08983E-08	0.4746	4.57800E-12	−0.3592	5.92433E-07	Promotes proteasomal degradation. Involved in transmembrane transport, cell signaling, protein modification and ubiquitination.

**Table 3:** Results from *Kaplan Meier* analysis of pEVs proteins’ ability to predict the conversion to AD dementia. Table shows the proteins that exhibited significance on the analysis.

Comparison of Survival Curves	CHMP1A	MAX	CCS	IL32
**Log-rank (Mantel-Cox) test**				
**Chi square**	5.45	4.145	6.721	4.73
**P value**	0.0196	0.0417	0.0095	0.0296
**P value summary**	*	*	**	*
**Are the survival curves sig different?**	Yes	Yes	Yes	Yes
**Median survival (years)**				
**High levels**	2.453	2.283	2.283	2.448
**Low levels**	5.413	5.046	5.243	5.046
**Hazard Ratio (Mantel-Haenszel)**	Low/High levels	Low/High levels	Low/High levels	Low/High levels
**Ratio**	2.234	2.04	2.488	2.074
**95% CI of ratio**	1.138 to 4.388	1.027 to 4.054	1.249 to 4.956	1.075 to 4.001

## Data Availability

The data that support the findings of this study are available from the corresponding authors upon a reasonable request and a data transfer agreement.

## Highlights

- Patients with AD dementia showed an enhanced biogenesis of pEVs.
- A wide variety of pEVs proteins, previously described to be involved in AD, significantly correlated with their CSF homologous, whilst serums did not.
- pEVs proteome showed a specific AD-related signature in MCI A + T + patients.
- Increased pEVs protein levels strongly correlated with common AD features such as reduced CSF Aβ42 and increased CSF pTau181.
- Increased pEVs protein levels were associated to lower scores on cognitive tests.
- pEVs proteins exhibited high correlations with brain ventricles, and CSF volume in MRI analysis.
- Several neurology-related pEV proteins predicted conversion from MCI to AD dementia.
- CHMP1A levels in pEVs showed the best diagnostic accuracy of AD pathology.

## Abbreviations

Aβ: amyloid β
AD: Alzheimer’s disease
BBB: blood-brain barrier
CDR: Clinical Dementia Rating
CNS: central nervous system
cryo-TEM: cryo-transmission electron microscopy
CSF: cerebrospinal fluid
DALYs: disability- adjusted life-years
EVs: extracellular vesicles
FU: follow-up
pEVs: plasma extracellular vesicles
PRS: polygenic risk score
LP: lumbar puncture
MCI: mild cognitive impairment
NPX: normalized protein expression
NTA: Nanoparticle Tracking Analysis
PET: positron emission tomography
pTau: hyperphosphorylated tau
tTau: total Tau
UC: ultracentrifugation
WM: white matter
